# Pelvic and hypogastric nerves are injured in a rat prostatectomy model, contributing to development of stress urinary incontinence

**DOI:** 10.1038/s41598-018-33864-3

**Published:** 2018-11-06

**Authors:** Marah Hehemann, Shawn Choe, Elizabeth Kalmanek, Daniel Harrington, Samuel I. Stupp, Kevin T. McVary, Carol A. Podlasek

**Affiliations:** 1 Department of Urology, University of Illinois at Chicago, Chicago, IL 60612 USA; 2 Department of Urology, Loyola University Stritch School of Medicine, Maywood, IL 60153 USA; 3 UTHealth, The University of Texas Health Science Center at Houston, Department of Diagnostic and Biomedical Sciences, Houston, TX 77054 USA; 4 Simpson Querrey Institute, Department of Chemistry, Department of Materials Science and Engineering, and Biomedical Engineering, Northwestern University, Feinberg School of Medicine, Chicago, IL 60611 USA; 5 Departments of Urology, Physiology and Bioengineering, University of Illinois at Chicago, Chicago, IL 60612 USA

## Abstract

Urinary incontinence affects 40% of elderly men, is common in diabetic patients and in men treated for prostate cancer, with a prevalence of up to 44%. Seventy-two percent of prostatectomy patients develop stress urinary incontinence (SUI) in the first week after surgery and individuals who do not recover within 6 months generally do no regain function without intervention. Incontinence has a profound impact on patient quality of life and a critical unmet need exists to develop novel and less invasive SUI treatments. During prostatectomy, the cavernous nerve (CN), which provides innervation to the penis, undergoes crush, tension, and resection injury, resulting in downstream penile remodeling and erectile dysfunction in up to 85% of patients. There are other nerves that form part of the major pelvic ganglion (MPG), including the hypogastric (HYG, sympathetic) and pelvic (PN, parasympathetic) nerves, which provide innervation to the bladder and urethra. We examine if HYG and PNs are injured during prostatectomy contributing to SUI, and if Sonic hedgehog (SHH) regulatory mechanisms are active in the PN and HYG nerves. CN, PN, HYG and ancillary (ANC) of uninjured, sham and CN crush/MPG tension injured (prostatectomy model) adult Sprague Dawley rats (n = 37) were examined for apoptosis, sonic hedgehog (SHH) pathway, and intrinsic and extrinsic apoptotic mechanisms. Fluorogold tracing from the urethra/bladder was performed. PN and HYG response to SHH protein was examined in organ culture. TUNEL, immunohistochemical analysis for caspase-3 cleaved, -8, -9, SHH, Patched and Smoothened (SHH receptors), and neurite formation, were examined. Florogold positive neurons in the MPG were reduced with CN crush. Apoptosis increased in glial cells of the PN and HYG after CN crush. Caspase 9 was abundant in glial cells (intrinsic), while caspase-8 was not observed. SHH and its receptors were abundant in neurons and glia of the PN and HYG. SHH treatment increased neurite formation. PN and HYG injury occur concomitant with CN injury during prostatectomy, likely contributing to SUI. PN and HYG response to SHH treatment indicates an avenue for intervention to promote regeneration and prevent SUI.

## Introduction

Stress urinary incontinence (SUI) affects 40% of elderly men^1^, is common in diabetic patients^2^ and in men treated for prostate cancer, with a prevalence of up to 44%^3^. Seventy-two percent of prostatectomy patients develop SUI in the first week after surgery and individuals who do not recover within 6 months generally do no regain function without intervention. Incontinence has a profound impact on the physical and mental health of patients^1^, who view incontinence pad use as detrimental to their quality of life^4^. The artificial urinary sphincter (AUS) is the gold standard for the treatment of this disorder, however most men will continue to need at least one pad per day, and device failure, erosion of the urethra, urinary retention, transient pain and infection are significant side effects that lead to a revision rate of up to 80% by 10–15 years^5–7^. Thus, a critical unmet need exists to develop novel and less invasive SUI treatments/preventions.

**Figure 1:**
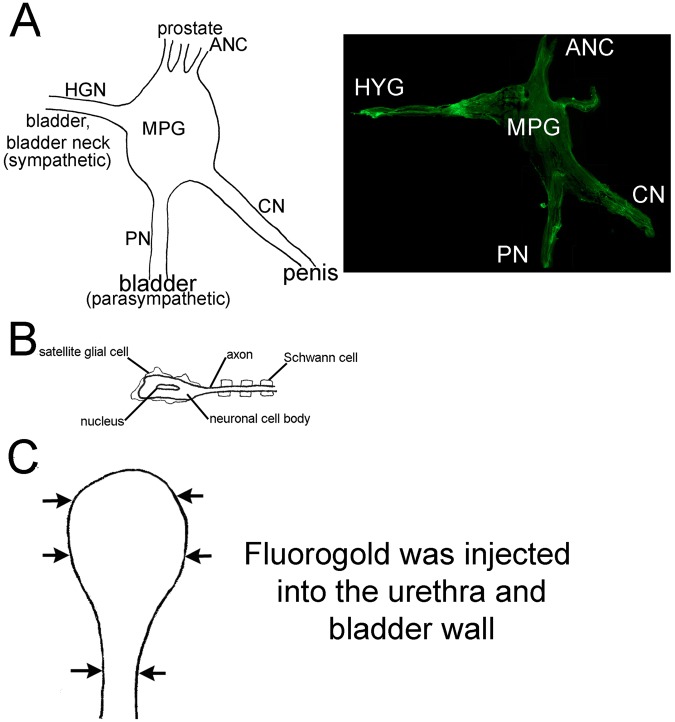
(**A**) Rat pelvic plexus. CN = cavernous nerve. PN = pelvic nerve. MPG = pelvic ganglia. HYG = hypogastric nerve. ANC = accessory nerves. (**B**) Diagram of a neuron including the cell body, nucleus, axon, Schwann cells and satellite glial cells. (**C**) Diagram of fluorogold injection into the wall of the bladder and urethra (arrows).

During prostatectomy, the cavernous nerve (CN), which provides innervation to the penis, undergoes crush, tension, and resection injury, resulting in downstream penile remodeling and erectile dysfunction (ED) in up to 85% of patients^8,9^. There are other nerves that form part of the major pelvic ganglion (MPG), including the hypogastric (HYG, sympathetic) and pelvic (PN, parasympathetic) nerves, which provide innervation to the bladder and urethra (Fig. 1A). The HYG controls bladder neck contraction and bladder relaxation while the PN regulates contraction of the bladder and opens the bladder neck to expel urine. Each nerve contains neurons, and glial cells which control the microenvironment, providing support, nutrients and receptors for signaling and communication (Fig. 1B). We hypothesize that other parts of the MPG including the HYG and PNs are injured during prostatectomy, likely due to tension injury on the MPG, and contribute to the development of post prostatectomy SUI. This idea is novel since it has been presumed that surgical removal of rhabdosphincter muscle, which occurs when the bladder is disconnected from the urethra and then reconnected after prostate removal, is the cause of SUI. However, preoperative erectile function predicts post-prostatectomy continence^10,11^, SUI recovery at 3 and 6 months correlates with neurovascular bundle sparing^12,13^, and a transient decrease in bladder compliance, capacity, leak point and increased non-voiding contractions were observed in a rat prostatectomy model^14^. In this study we examine the hypothesis that prostatectomy induced injury to the MPG extends beyond the CN, to the PN and HYG, and contributes to SUI.

A role for the Sonic hedgehog (SHH) pathway in PN and HYG homeostasis and regeneration has not previously been examined, but is critical for novel SUI therapy development, and to our understanding of if SHH is a global regulator of peripheral nerve homeostasis/regeneration. The CN forms part of the MPG along with the PN and HYG, and we’ve shown previously that SHH is essential to maintain CN morphology and function^15,16^. When the CN is injured, as occurs during prostatectomy, SHH protein decreases in the CN, and the neurons undergo apoptosis^16^. This is identical to what happens when SHH is inhibited in the CN, with resulting demyelination of myelinated fibers and axonal degeneration of non-myelinated fibers^15^. Loss of innervation from SHH inhibition also affects the morphology and function of the penis, resulting in substantially increased smooth muscle apoptosis and ED^17^. We successfully adapted a peptide amphiphile nanofiber hydrogel (PA) for SHH protein delivery to the CN that effectively accelerates regeneration, is neuroprotective, prevents apoptosis of penile projecting neurons, and improves erectile function ~60% at 6 weeks after injury^15,18^. If similar SHH signaling mechanisms are active in the PN and HYG, then SHH PA may be optimized to preserve/regenerate the PN and HYG, thereby reducing SUI development. In this study we examine SHH pathway signaling in all nerves of the MPG and examine the time dependent injury response (apoptosis) in a rat prostatectomy model.

## Methods

### Animals

Adult Sprague Dawley rats (n = 41, P115-120) were obtained from Charles River. The study was carried out in accordance with the recommendations in the Guide for the Care and Use of Laboratory Animals of the National Institutes of Health, and the experiments comply with the current laws of the country in which they were performed. The animal care protocol was approved by the Office of Animal Care and Institutional Biosafety at the University of Illinois at Chicago, and animals were cared for in accordance with institutional OACIB approval.

### Bilateral CN crush/MPG tension injury

Bilateral CN crush was performed on Sprague Dawley rats as previously described^16^. Briefly, MPG/CN were exposed and microforceps (size 0.02 × 0.06 mm) were used to crush the CN bilaterally for 30 seconds. This method of CN crush is commonly used in the literature^19,20^, and in our laboratory^15,16^. The extent and reproducibility of crush was previously verified in our laboratory^15^ with visible change in nerve color and indentation. Rats were sacrificed at 1, 2, 4 and 7 days after CN crush (n = 3, 3, 4, and 4) and the CN, PN, HYG, and ANC were dissected. Normal adult Sprague Dawley rats (n = 13), and sham controls (n = 6, CN exposed but not manipulated), were examined for comparison.

### Fluorogold injection into urethra and bladder wall

Sprague Dawley rats underwent sham (n = 2) or CN crush (n = 2) prior to fluorogold (5%w/v) injection into the wall of the urethra and bladder using a 10 μl Hamilton syringe with a 28-gauge needle (Restek). Three fluorogold injections were performed on each side (Fig. 1C). Two injections of 2.5 μl were performed into the bladder wall and one 5 μl injection into the wall of the bladder neck/urethra, while avoiding injection into the bladder lumen. Injection sites were sealed with glue (n-butyl cyanoacrylate). Rats were sacrificed after 7 days and the MPG were isolated.

### Immunohistochemical analysis (IHC)

IHC was performed on frozen CN, PN, HYG, and ANC nerves (n = 8 each) that were sectioned 14 µm in thickness. OCT was removed with 1XPBS prior to blocking in 3% milk. Sections were incubated overnight at 4 °C with 1/100 goat polyclonal antibodies for SHH (N19) and PTCH1 (Santa Cruz), and rabbit smoothened (SMO, LifeSpan BioSciences), caspase 3 cleaved and caspase 9 (Cell Signaling), and mouse caspase 8 (Cell Signaling). Secondary antibodies were chicken anti-goat, chicken anti-rabbit, and goat anti-mouse 594 (Molecular Probes). Control slides in which the primary antibody was omitted, were performed for all secondary antibodies, to ensure artifact staining was not present from the secondary antibodies. Sections were mounted using DPX Mounting media and fluorescence was visualized using a Leica DM2500 microscope.

### Tunel

Apoptotic cells were identified using the Apoptag kit (Millipore) on sham (n = 6) and 1, 2, 4, and 7 day CN crushed (n = 14) Sprague Dawley rat MPG, as previously described^21^. Fluorescence was visualized using a Leica DM2500 microscope.

### Organ culture

Bilateral CN crush was performed and MPG tissues (n = 4) were isolated after two days, and were embedded in sterile culture plates containing 150 μl of reduced growth factor Matrigel (Corning Life Sciences). Affi-Gel beads (100–200 mesh, Bio Rad), incubated overnight at 4 °C with 1XPBS (n = 2) or SHH protein (25 μl of a 1 μg/μl solution, R&D Systems, n = 2), were embedded in the Matrigel near the MPG. Matrigel was gelled at 37 °C for 5 minutes prior to adding RPMI media (Sigma) and an antibiotic cocktail (100X Penicillin-Streptomycin-Glutamine, Thermo Fisher). Culture plates were placed in an atmosphere controlled incubator (5% CO_2_) at 37 °C for three days.

## Results

### Interruption of innervation between the urethra/bladder neck/bladder and MPG with CN crush

**Figure 2:**
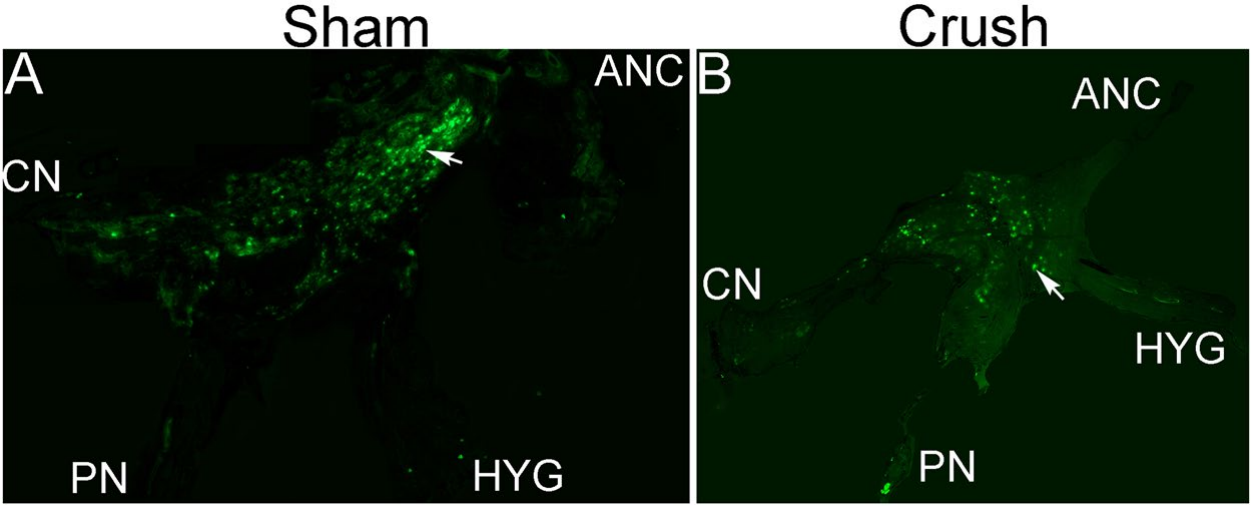
Fluorogold was injected into the bladder and urethral wall of sham and CN crushed Sprague Dawley rats. After 7 days the MPG were isolated and examined for fluorogold, which underwent retrograde transport to neurons of the MPG that innervate the bladder and urethra, and composite photos of all nerves and MPG were assembled from 100X photos. (**A**) Sham MPG show many fluorogold staining neurons (arrows), indicating intact innervation. (**B**) Fluorogold stained neurons are reduced in the MPG with CN crush/MPG tension injury, indicating interruption of innervation between the MPG and bladder/urethra.

Fluorogold was abundant in neurons dispersed throughout the MPG in sham rats (Fig. 2A), indicating intact innervation. After CN crush, the number of fluorogold positive neurons decreased throughout the MPG (Fig. 2B), including regions that innervate the bladder and urethra, indicating interruption of innervation to the urethra/bladder neck/bladder occur with MPG tension/CN crush injury.

### Apoptosis under normal, homeostatic conditions

**Figure 3:**
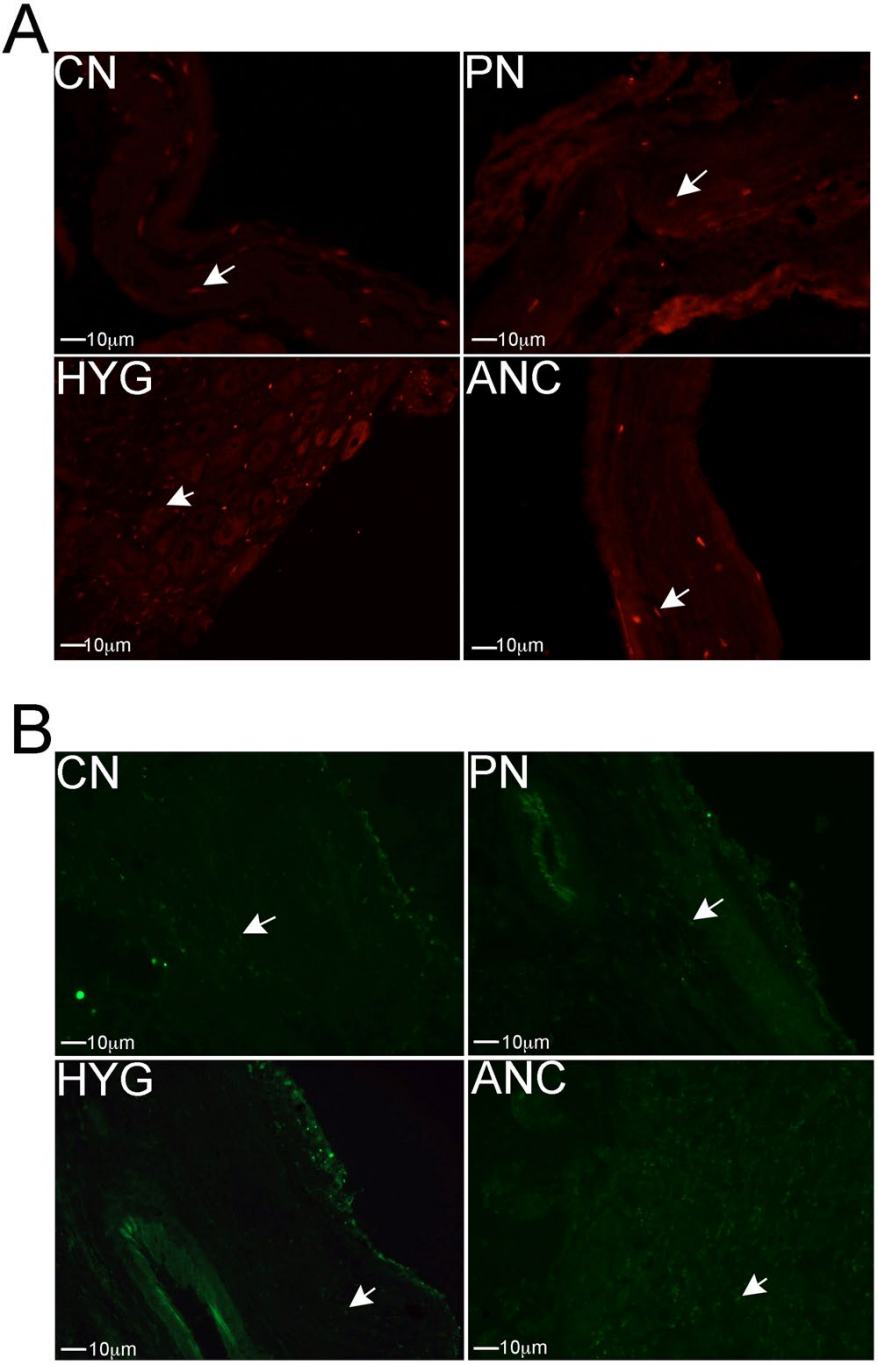
(**A**) Immunohistochemical analysis for caspase 3 cleaved protein (apoptotic marker) in the MPG of rats that under went sham surgery, where the MPG was exposed but the nerves were uninjured. Caspase 3 cleaved protein was present at a low level in the CN, PN, HYG and ANC nerves, indicating a low level of apoptosis under normal homeostatic conditions. Arrows indicate caspase 3 cleaved protein. 100X magnification. (**B**) TUNEL assay of the sham rat MPG confirms a low level of apoptosis in all nerves of the MPG. Arrows indicate apoptotic cells. 100X magnification.

IHC was performed assaying for caspase 3 cleaved protein, a marker of apoptotic cells, in MPG from rats that underwent sham surgery. Caspase 3 cleaved was identified at a low level in the CN, PN, HYG, and ANC nerves (Fig. 3A), indicating low cell turnover under uninjured homeostatic conditions. Low abundance apoptosis was confirmed by TUNEL analysis (Fig. 3B) of sham MPG.

### Apoptosis in MPG nerves with CN crush/MPG tension injury

**Figure 4:**
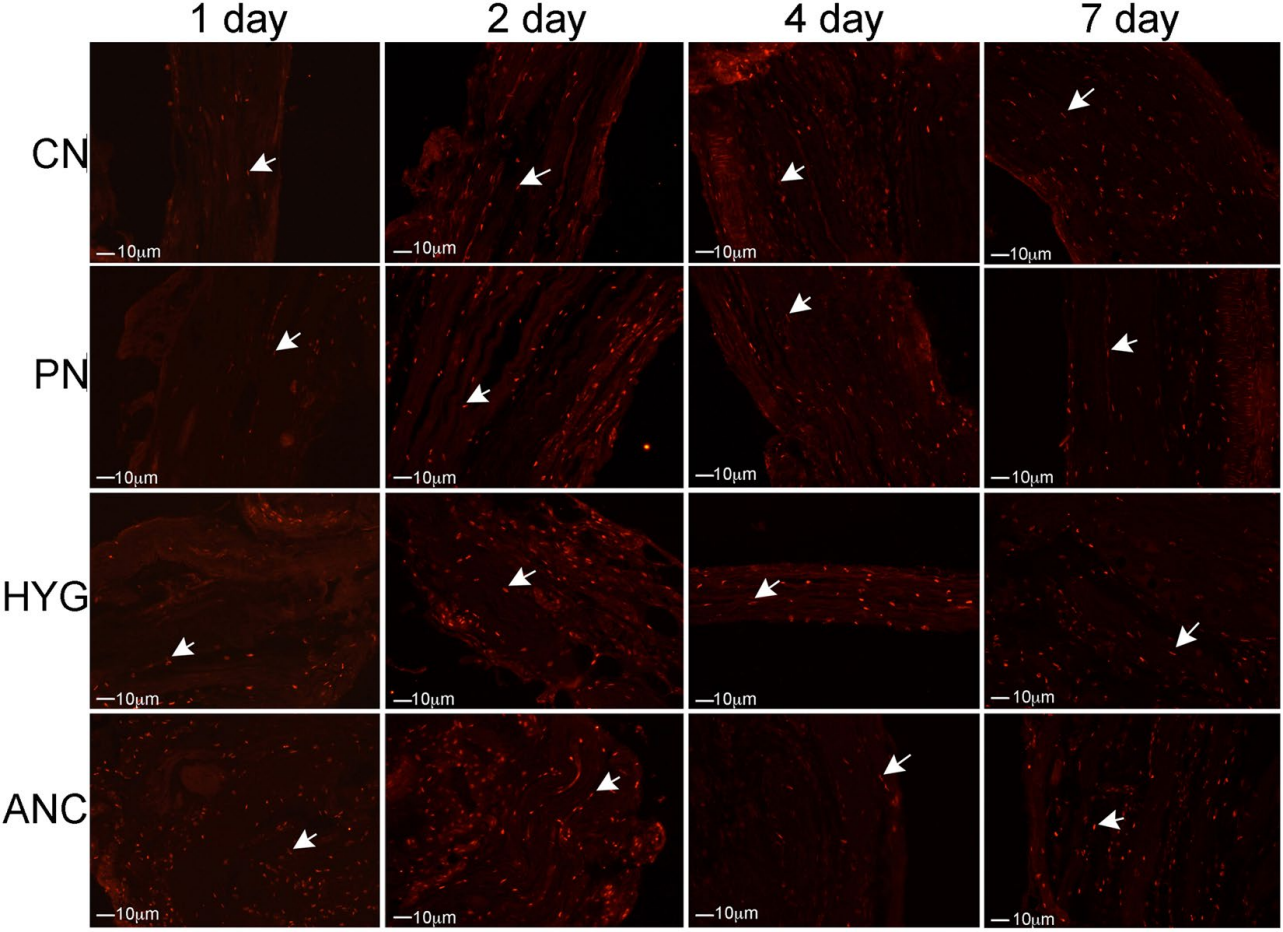
Immunohistochemical analysis for caspase 3 cleaved protein in the CN, PN, HYG, and ANC nerves of the rat MPG, 1–7 days after bilateral CN crush/MPG tension injury. Abundant caspase 3 protein was observed in all of the MPG nerves, in a time dependent manner, in response to CN crush. Staining appeared primarily in glial cells of the MPG and Schwann cells of the nerves. Arrows indicate caspase 3 cleaved protein. 200–400X magnification.

**Figure 5:**
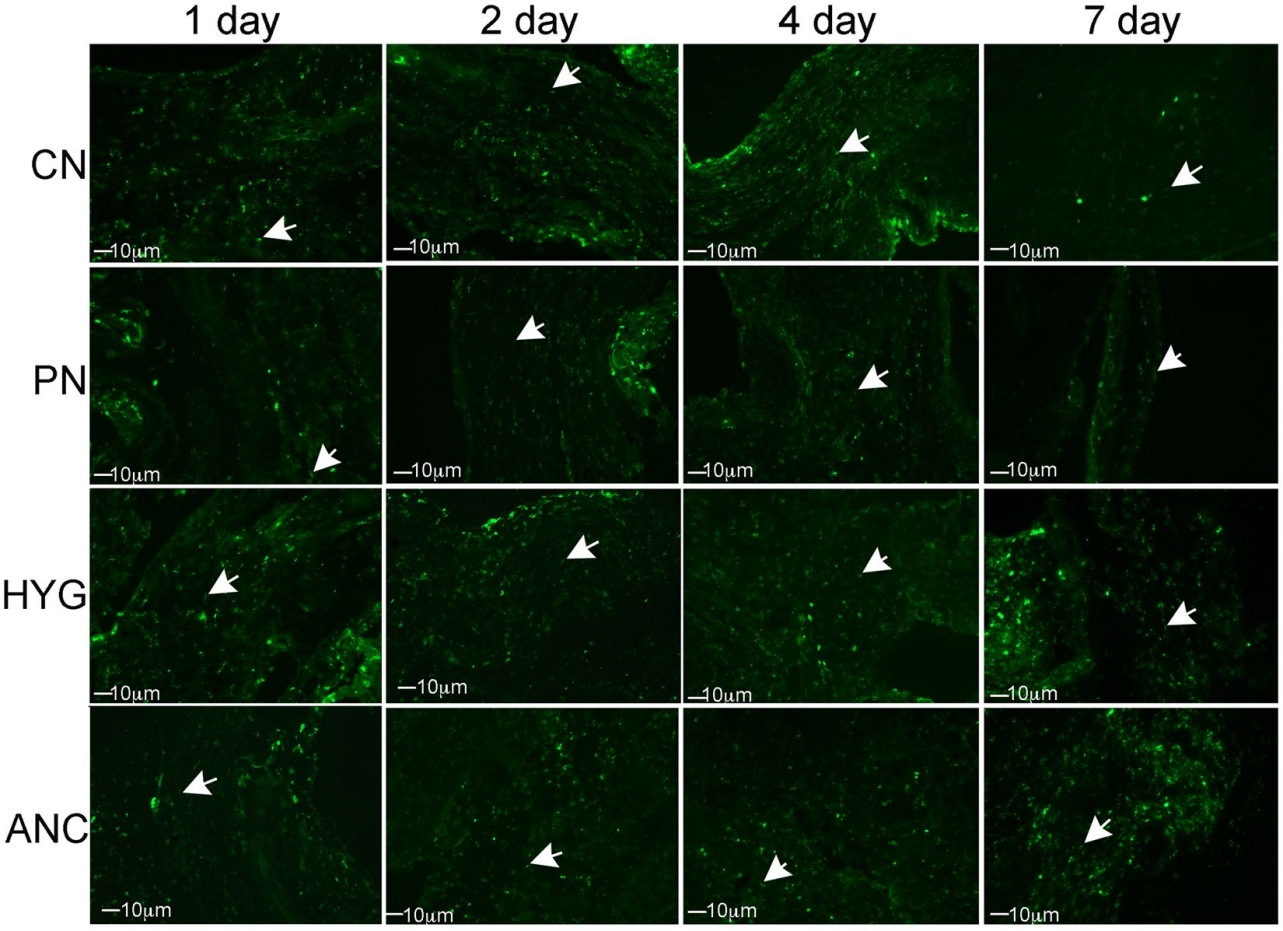
TUNEL assay of CN, PN, HYG, and ANC nerves 1–7 days after CN crush injury. Apoptosis appeared primarily in glial/Schwann cells of all nerves in a time dependent manner after CN crush injury. Arrows indicate apoptotic cells. 200–400X magnification.

IHC was performed on rat MPG 1–7 days after bilateral CN crush. Caspase 3 cleaved protein was up-regulated in a time-dependent manner, primarily in glial cells of the CN, PN, HYG, and ANC nerves from 1–7 days after CN crush/MPG tension injury (Fig. 4A). For a detailed analysis of glial and neuronal cell identification in the pelvic ganglia, and neuronal cells body versus axon, see Angeloni *et al*., 2013 and Choe *et al*.^16,22^. Caspase 3 cleaved was most abundantly observed at day 1 after injury in the ANC nerves, which provide innervation to the prostate. By day 2, caspase 3 cleaved was also abundant in the CN, PN and HYG nerves, and remained abundant at 4 and 7 days after injury (Fig. 4). Apoptosis was confirmed by TUNEL assay in glial cells of all nerves (Fig. 5).

### Apoptotic mechanism

**Figure 6:**
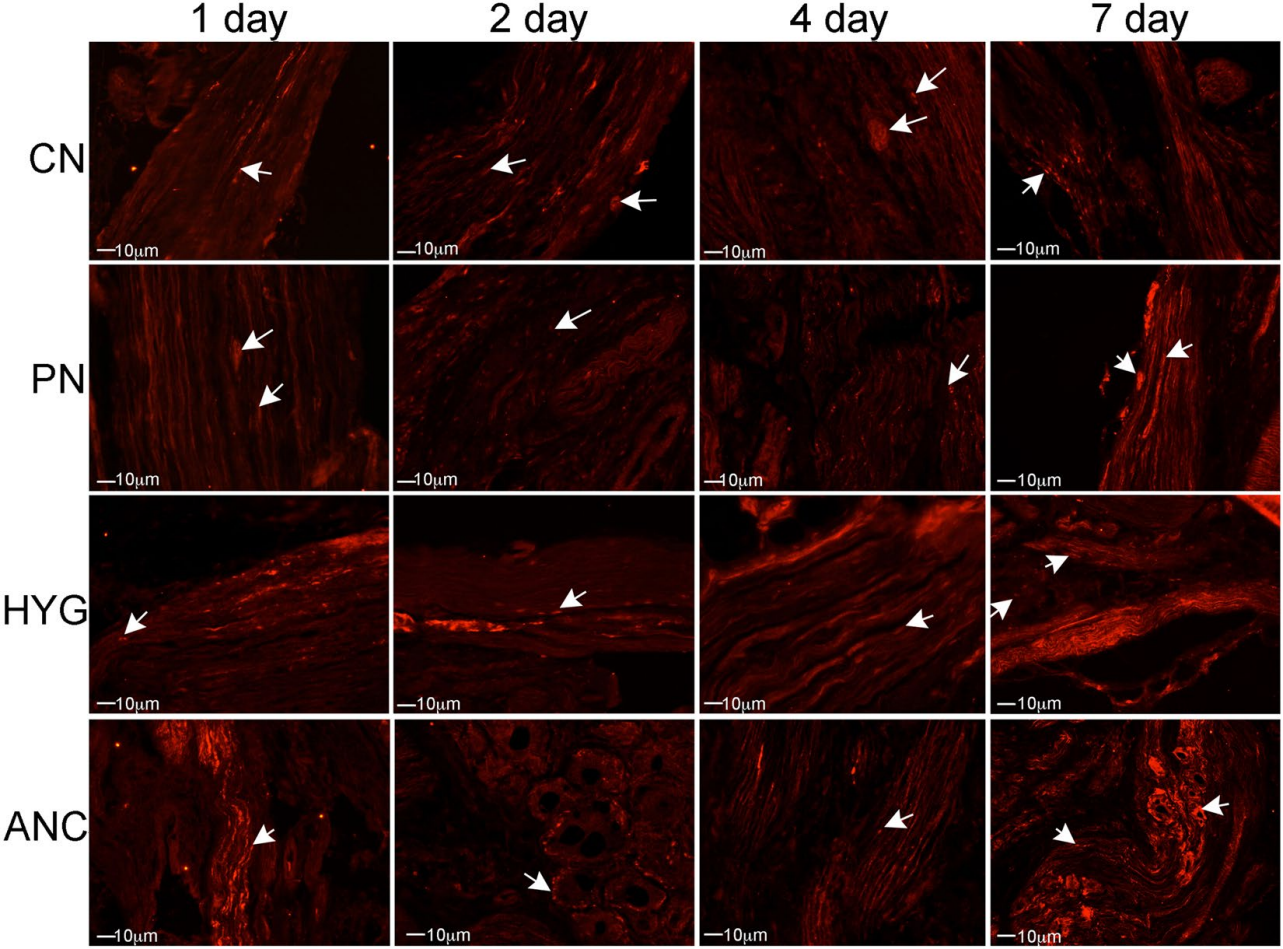
Immunohistochemical analysis of caspase 9 protein in MPG, CN, PN, and ANC nerves, 1–7 days after CN crush injury. Caspase 9 protein was identified primarily in glial/Schwann cells of all MPG nerves and in a small number of neurons, indicating that the mechanism of how apoptosis takes place in the pelvic plexus after injury is primarily through the intrinsic apoptotic pathway. Arrows indicate caspase 9 protein. 200–400X magnification.

**Figure 7:**
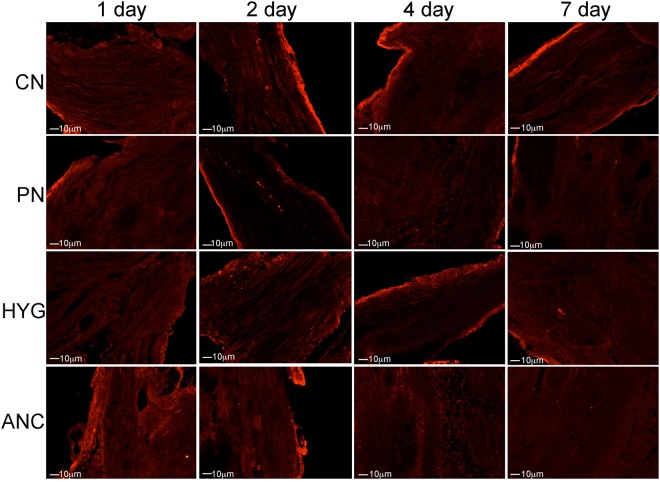
Immunohistochemical analysis of caspase 8 protein in MPG, CN, PN, and ANC nerves, 1–7 days after CN crush injury. Caspase 8 protein was absent in all pelvic plexus nerves, indicating the absence of extrinsic apoptotic pathway activity. 200–400X magnification.

We examined the pathway by which apoptosis takes place in the rat MPG after injury, by examining if caspase 9 and/or caspase 8 proteins were increased in the CN, PN, HYG and ANC nerves from 1–7 days after CN crush. Caspase 9 (intrinsic) was observed in all nerves of the MPG, and was primarily identified in Schwann cells of the CN, PN, HYG, and ANC nerves (Fig. 6). Only a small number of neurons stained for caspase 9 (Fig. 6), indicating that it is primarily glial cells/support cells that undergo apoptosis in the first week after CN injury. Caspase 8 (extrinsic) protein was not identified in any of the nerves of the MPG 1–7 days after CN injury (Fig. 7), indicating that the apoptotic mechanism occurs primarily through the intrinsic pathway.

### Sonic hedgehog pathway is abundant in all nerves of the uninjured MPG

**Figure 8:**
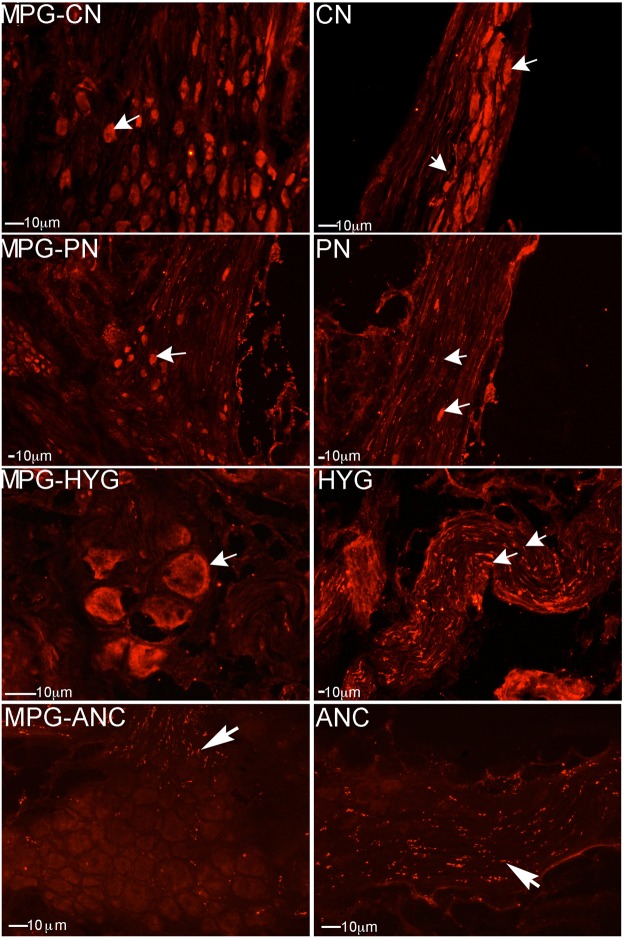
Immunohistochemical analysis of SHH protein in normal/uninjured MPG, CN, PN, HYG and ANC. SHH protein was abundant in MPG neurons that innervate the penis, bladder, and prostate and in neurons and glial cells of the CN, PN, HYG and ANC. Arrows indicate SHH protein. 100–200X magnification.

**Figure 9:**
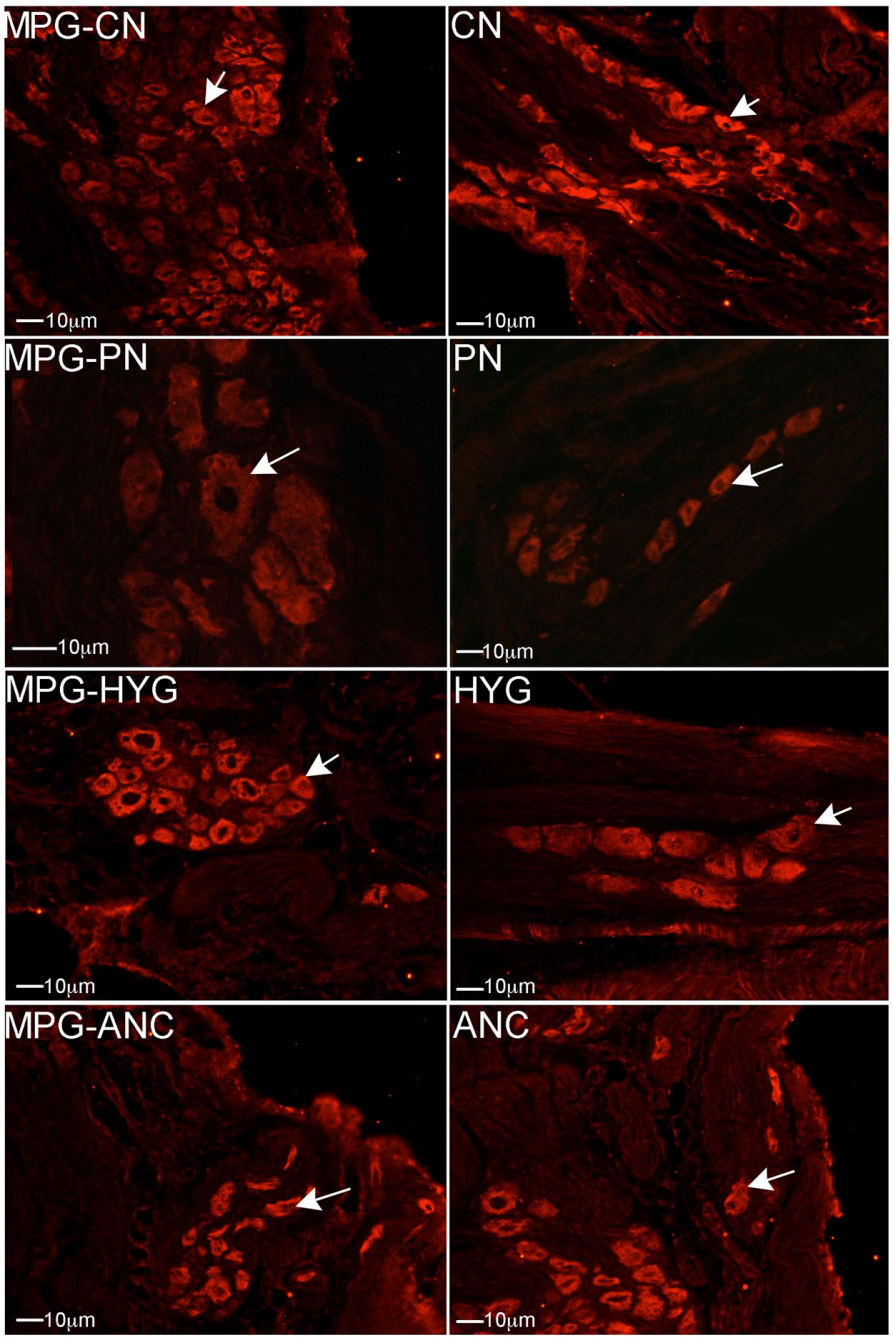
Immunohistochemical analysis of the SHH receptor PTCH1, in MPG from normal/uninjured rats. PTCH1 protein was abundant in neurons of the MPG that innervate the penis, bladder and prostate and the associated CN, PN, HYG and ANC nerves. PTCH1 was not identified in glial cells. Arrows indicate PTCH1 protein. 200–400X magnification.

**Figure 10:**
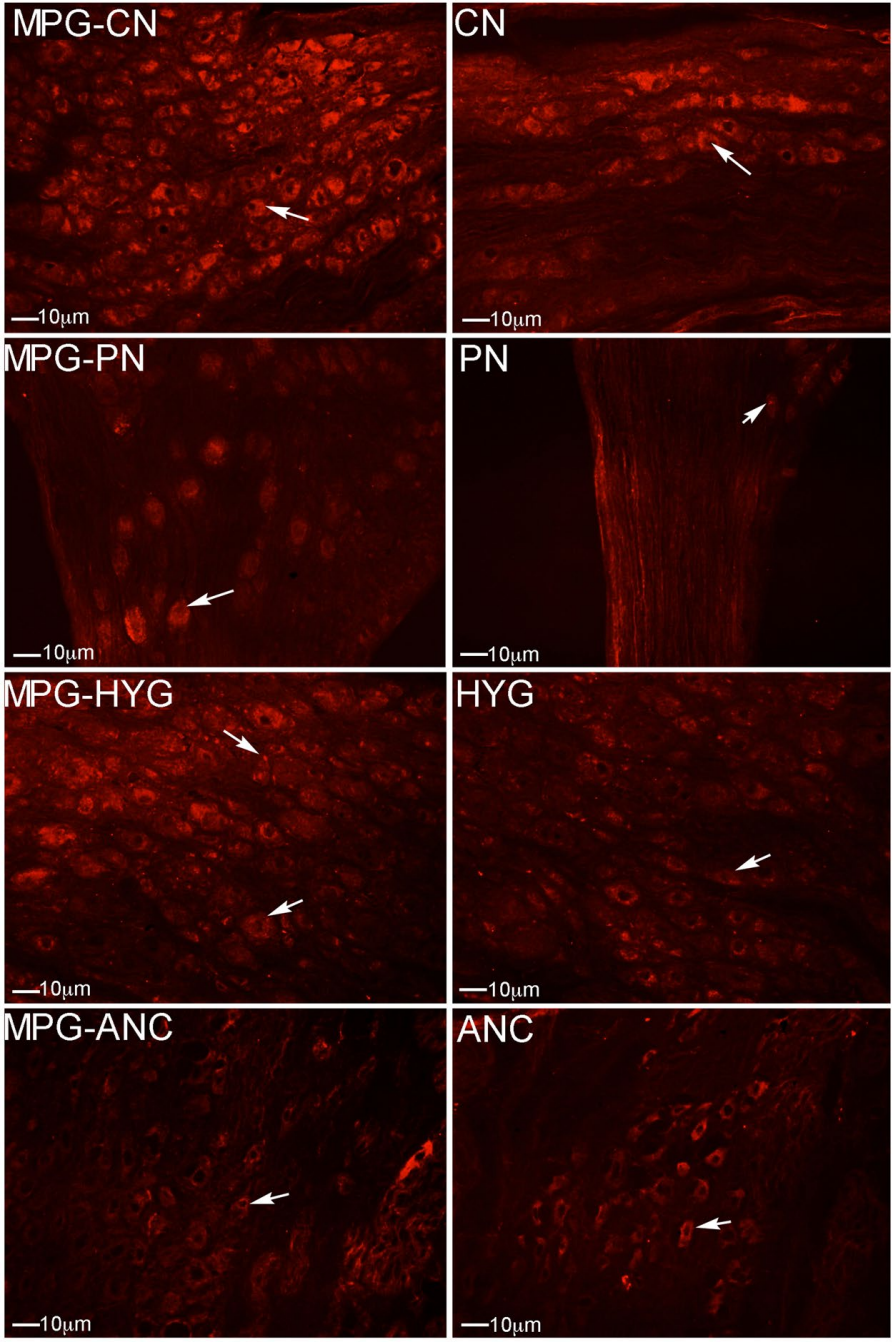
Immunohistochemical analysis of the SHH receptor SMO, in MPG from normal/uninjured rats. SMO was abundant in neurons of the MPG that innervate the penis, bladder, and prostate and in the associated CN, PN, HYG and ANC nerves. Arrows indicate SMO protein. 200–400X magnification.

IHC for SHH, and it’s receptors PTCH1 and SMO, were performed on normal/uninjured rat MPG isolated from Sprague Dawley rats (n = 8). SHH protein was identified in neurons of the caudal portion of the MPG that innervates the penis, and in neurons and Schwann cells of the CN (Fig. 8). SHH protein was abundant in the region of the MPG that innervates the PN and the HYG, and in neurons and glial cells of the PN and HYG (Fig. 8). SHH protein was also present in ANC neurons (Fig. 8). PTCH1 was identified in neurons of the MPG that provide innervation to the penis, bladder, and prostate, and the respective nerves (Fig. 9). PTCH1 was not observed in glial cells (Fig. 9). SMO was abundant in MPG regions that innervate all nerves of the MPG and in the respective nerves (Fig. 10). Both neurons and associated glial cells stain for SMO (Fig. 10).

### SHH protein treatment induces neurite formation in PN and HYG neurons

**Figure 11:**
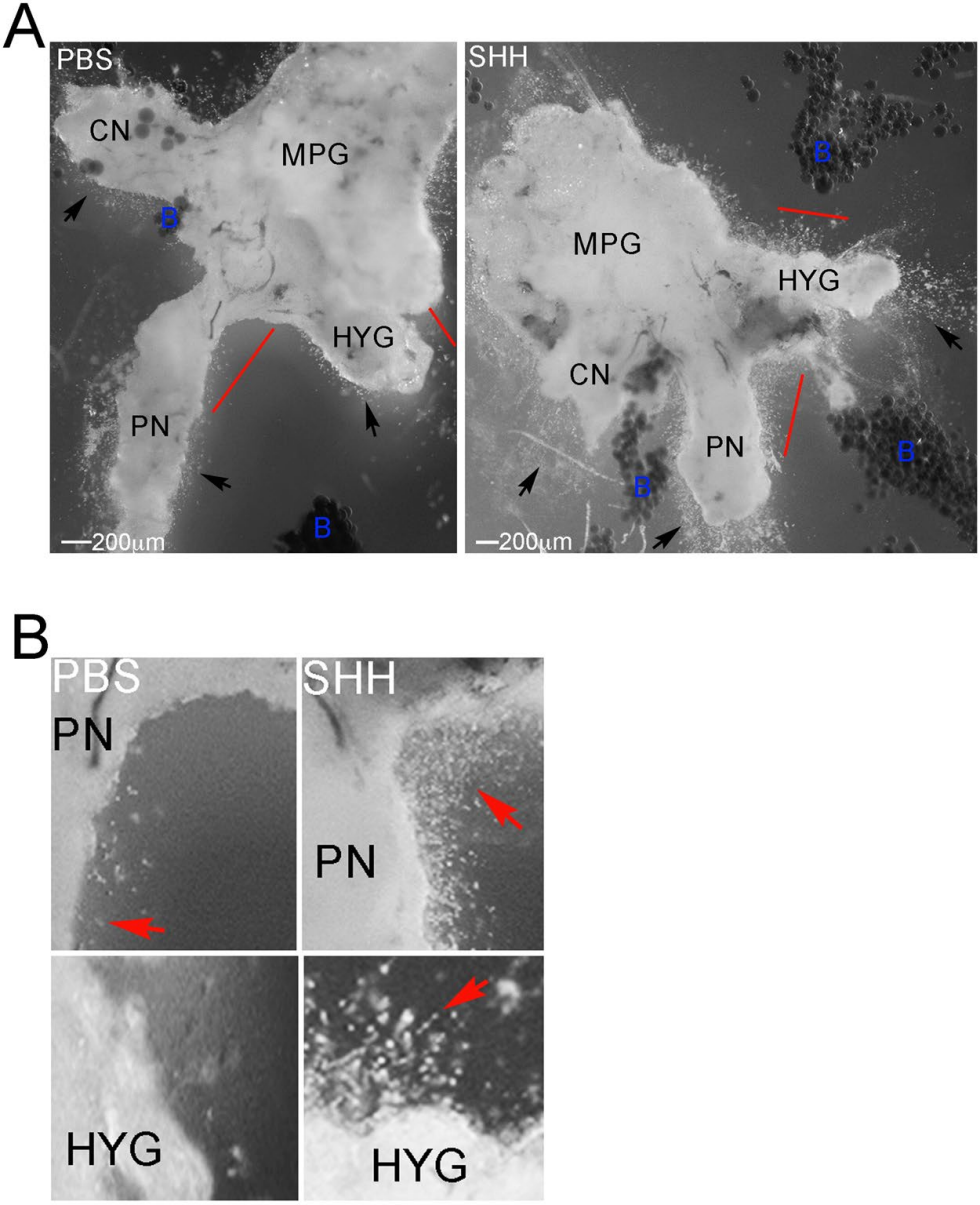
MPG from adult Sprague Dawley rats that underwent CN crush/MPG tension injury were dissected after two days and were grown in organ culture for three days with PBS or SHH protein. Low abundance neurite formation occurred from neurons in all nerves of the MPG with CN crush (**A**). SHH treatment increased the number of neurites that formed from all nerves after injury (**A**) indicating that PN and HYG neurons are responsive to SHH treatment. Arrows indicate neurites. Red bar is region expanded in B. 40X magnification. Enlarged regions of the PN and HYG show abundant neurite formation with SHH treatment in comparison to PBS controls (**B**).

MPG from Sprague Dawley rats that underwent CN crush/MPG tension injury were dissected after two days and grown in organ culture for three days with PBS or SHH protein. Low abundance neurite formation occurred from neurons in all nerves in response to CN crush (Fig. 11A,B). SHH treatment increased the number of neurites that formed from all nerves after injury (Fig. 11A,B), indicating that PN and HYG neurons are responsive to SHH treatment. We previously showed that uninjured MPG/CN undergoes very little neurite formation, which increases with SHH treatment^23^.

## Discussion

Unlike the penile projecting neurons of the CN, which cluster in the “horn” region of the MPG, bladder neurons are relatively evenly distributed throughout the ganglion^24^. When the CN is crushed, simulating the MPG tension injury which occurs during prostatectomy, there is an interruption of innervation between the bladder/bladder neck/urethra and the MPG, as is shown by the decrease in fluorogold positive neurons (Fig. 2). This previously unsuspected loss of innervation, may contribute to bladder dysfunction observed in men after prostatectomy. Injury to the entire MPG, including the PN and HYG nerves, was confirmed with IHC analysis of caspase 3 cleaved (apoptotic marker) and TUNEL assay. Apoptosis/cell turn over is normally low in the MPG under uninjured homeostatic conditions. However with CN crush/MPG tension injury, apoptosis increased in all nerves of the MPG in a time dependent manner, indicating that not only is the CN injured during prostatectomy, but also the PN and HYG, and thus may contribute to SUI development in rats following CN crush injury.

Apoptosis occurs primarily in glial cells of all nerves of the MPG in the first week after CN injury. Glial cells surround the neuronal cell body and control the microenvironment of sympathetic ganglia, providing support, nutrients and receptors for signaling and communication. It is suggested that the change in the neuronal microenvironment, caused by loss of growth factors and interaction provided by the glia, leads to later neuronal apoptosis, after the first week post-injury. We examined the mechanism of how apoptosis takes place in all nerves of the MPG. There are two apoptotic pathways that stimulate apoptosis via a caspase dependent mechanism^25,26^. These are the extrinsic pathway, which is initiated by a death ligand, and the intrinsic pathway, which is dependent on the mitochondria^27^ and formation of the apoptosome, which facilitates activation of procaspase 9 to the active form. Once activated, caspase 8 in the extrinsic pathway, and caspase 9 in the intrinsic pathway, cleave and activate downstream caspase 3 and 7, resulting in activation of several target proteins and ultimately apoptosis^28–30^. In our study we examined caspase 3 cleaved and performed TUNEL assay to identify apoptotic cells, and then examined caspase 8 and 9 in all nerves of the MPG, to determine the mechanism. Caspase 9 was identified in glia of CN, PN, HYG and ANC nerves from 1–7 days after CN injury. Caspase 8 was not identified in any of the nerves after CN injury, indicating that the apoptotic mechanism takes place through an intrinsic, mitochondrial dependent mechanism. This is significant since intervention to prevent the apoptotic response may be possible using localized delivery of caspase inhibitors to the MPG at the time of prostatectomy, and thus impact nerve function, and potentially SUI and ED.

This study is the first identification that the SHH pathway is active in the PN and HYG. SHH function within PN and HYG neurons and glia is supported by neurite activation in the presence of exogenous SHH protein, suggesting that SHH may be useful to promote PN and HYG regeneration. This is similar to our observations in the CN in which SHH protein decreases with injury, SHH treatment increases neurite formation of penile projecting neurons, and SHH treatment using PA nanofiber hydrogels was neuroprotective, promoted CN regeneration and improved erectile function in a rat prostatectomy model^15,16^. Our findings suggest that the PA we adapted for SHH delivery to the CN to promote regeneration and improve erectile function, may be useful with optimization for delivery to the PN and HYG at the time of prostatectomy, to protect the nerves from injury, promote regeneration, and potentially improve SUI treatment options.

## Conclusions

Prostatectomy causes injury to the PN and HYG nerves, suggesting a mechanism of how SUI develops, and the SHH pathway is an important mediator of PN and HYG nerve homeostasis with potential to enhance regeneration and prevent SUI.

## Data Availability

All data generated or analyzed during this study are included in this published article.
